# Immune interplay between sepsis and haematological malignancies

**DOI:** 10.1186/s40635-026-00904-6

**Published:** 2026-04-28

**Authors:** Stéphanie Pons, Morgane Grosset, Emmanuelle Touze, Lara Zafrani

**Affiliations:** 1 UMR 1342, INSERM, Institut de recherche Saint-Louis, Université Paris Cité, Paris, France; 2 Anesthésie-Réanimation, Hôpital Avicenne, Hôpitaux Universitaires Paris Seine-Saint-Denis, Assistance Publique-Hôpitaux de Paris, Bobigny, France; 3 Medical Intensive Care Unit, Saint-Louis Hospital, Assistance Publique-Hôpitaux de Paris, 1 Avenue Claude Vellefaux, 75010 Paris, France

**Keywords:** **Keywords**, Sepsis, Haematological malignancies, Acute myeloid leukemia, Immunosuppression

## Abstract

Haematological malignancies (HMs) are characterized by profound immune dysregulation, which increases susceptibility to sepsis. This dysregulation arises from the underlying disease and may be exacerbated by treatments. Sepsis itself induces a biphasic immune response, with an initial hyperinflammatory phase followed by prolonged immunosuppression that can persist for months or years, potentially impacting the course of HMs. This review examines the reciprocal interactions between HMs and sepsis, focusing on innate and adaptive immune alterations, the influence of malignancy-associated dysbiosis, and insights gained from experimental models. In addition, we discuss how sepsis-induced systemic inflammation may influence the progression of the underlying malignancy, through pro-inflammatory cytokine signaling and alterations in the bone marrow microenvironment. Understanding these complex interactions may provide a framework for mitigating infection-related complications and for exploring their influence on the natural history and progression of HM.

## Introduction

To date, 6,5% of all cancers worldwide are haematological malignancies (HMs), including non-Hodgkin (NHL) and Hodgkin lymphomas, leukemias, and multiple myeloma [1]. Major improvements in treatments and care over the past decades have overall increased 5 years to long term survival in patients with HMs [2]. Concurrently, their need for intensive care unit (ICU) admission significantly increased throughout the course of the disease: up to 15% patients with acute myeloid leukemia (AML) will require an hospitalization in the ICU [3], negatively impacting patients’ outcomes [4]. Sepsis, as well as acute respiratory failure, are the leading life-threatening conditions requiring ICU admission in this population, and is still associated with high rates of mortality [5]. Immunosuppression and immunomodulation are hallmarks of HMs and may be both due to the underlying disease or therapies (chemotherapy, immunotherapy or hematopoietic stem cell transplant), increasing the risk for sepsis and septic shock. On the other hand, sepsis induces an initial hyperinflammatory phase, concomitantly to a profound immunosuppression state that can persist for months or years following the acute infection, which may impact the natural history of HMs.

This review discusses the immune interplay between sepsis and HMs focusing on the innate and adaptive immune dysregulation induced by the malignancies, and the potential role of sepsis on HMs evolution and progression. The aim of this review is to synthesize emerging clinical and experimental observations to explore potential bidirectional interactions between sepsis-associated immune dysregulation and HM biology, rather than to establish causality. Articles were identified through PubMed searches using relevant keywords (e.g., « sepsis», « immune dysregulation», « hematologic malignancy», « acute myeloid leukemia», « lymphoma») and by reviewing reference lists of key publications. We focused on studies addressing the pathophysiological interactions between sepsis and HMs.

## Haematological malignancy-induced immune dysregulation and its impact on sepsis

HMs comprise a wide range of diseases, each one of them displaying particular effects on the innate and adaptive immune cells, both systemically and in the tumor microenvironment, increasing the occurrence of severe infections and organ failure. As acute myeloid leukemia (AML), acute lymphocytic leukemia (ALL) and lymphoma patients present a higher risk of ICU admission, we chose to focus on these specific HMs in this review [6]. While therapy-related-complications, including immunological effects of chemotherapies [7] and immunotherapies [8] contribute significantly to sepsis risk in patients with HM, these effects have been reviewed elsewhere and will not be discussed here.

### Innate immune dysregulation

Apart from chemotherapy-induced neutropenia, it has been shown that HMs could have a direct effect on innate immune cell phenotype and function, increasing the risk for infection and tumor progression. Indeed, in AML patients, neutropenia can also arise from underlying bone marrow dysfunction. In both AML mouse and human bone marrow neutrophils, Goel et al. demonstrated that the AML microenvironment could alter neutrophil maturation through nuclear factor κB (NF-κB) signaling and that these neutrophils expressed CD14 and participated in the tumor microenvironment immunosuppression by suppressing the proliferation of CD8 + T cell [9]. Moreover, a high neutrophil-to-lymphocyte ratio was found to be significantly correlated to poor overall survival in relapsed or refractory AML [10]. In diffuse large B-cell lymphoma (DLBCL), Shree et al. established in a large retrospective cohort of 21,690 survivors of DLBCL that they had an increased risk of immune-related conditions compared to survivors of solid cancers [11]. Notably, higher incidence rates of fungal pneumonia, viral pneumonia and meningitis were observed. Interestingly, these increased risk persisted in sensitivity analyses controlling for exposure to chemotherapy or stem cell transplantation [11]. These findings corroborate interesting data demonstrating that DLBCL altered the immune imprint for years following remission and seemed not to be influenced by therapy, leading the authors to suggest that cancers could imprint a disease-specific “immune scar”, which could impair for a long time innate and adaptive immune responses [12].

**Fig. 1:**
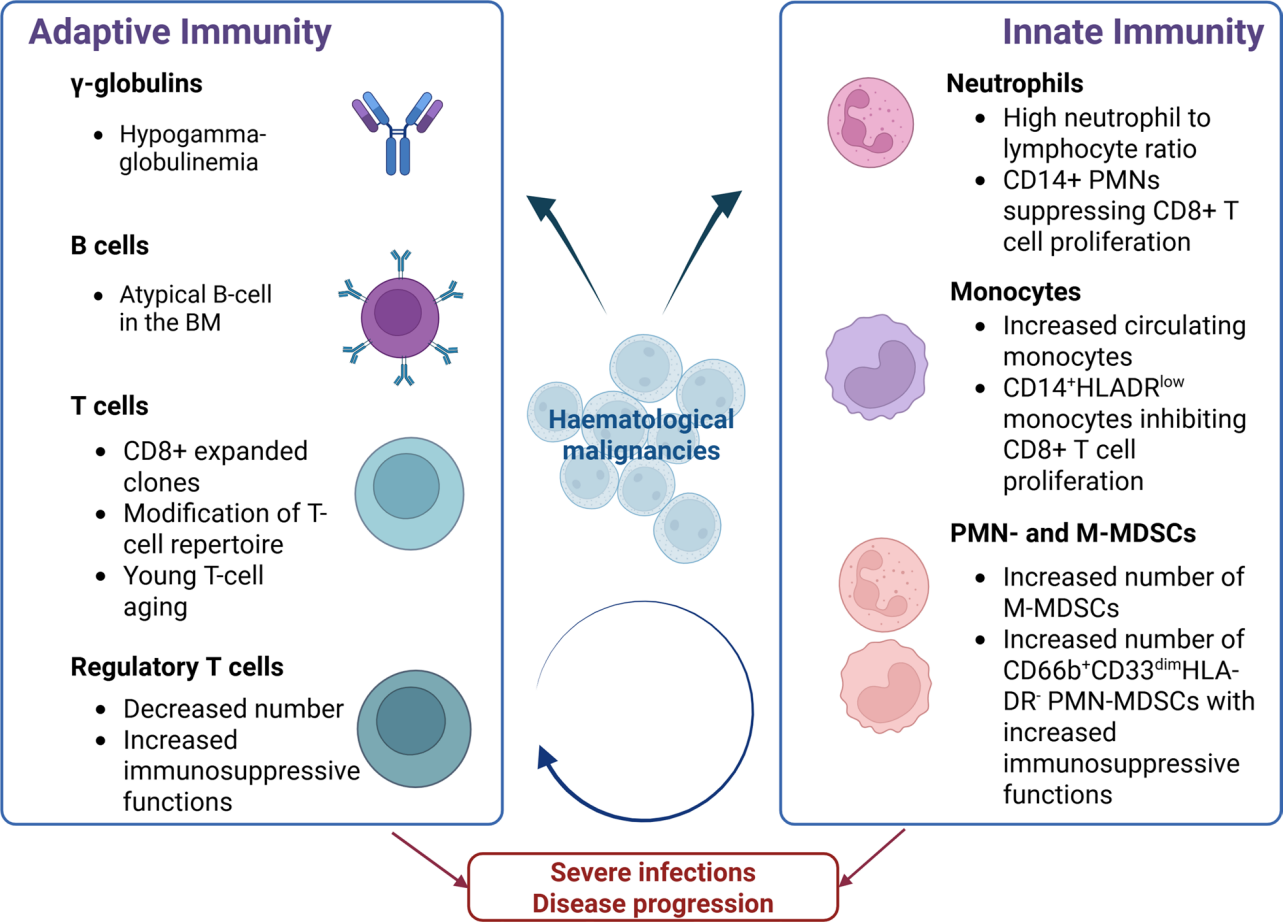
Mechanisms of haematological malignancy-induced immune dysregulation. M-MDSC: Monocytic myeloid-derived suppressor cells; PMN-MDSC: Granulocytic myeloid-derived suppressor cells. Created in BioRender. Pons, S. (2026) https://BioRender.com/u5w2iau

Neutrophils are not the only innate immune cells to be altered by HMs. In a mouse model of B-cell NHL, McKee et al. demonstrated that there was an increase in circulating monocytes. Although both LY6C^high^ and Ly6C^low^ monocytes populations were found in the blood of B-cell lymphoma mice, Ly6C^low^ monocytes differentially expressed immunosuppressive genes and suppressed CD8 + T cell activity using soluble factors as Indoleamine 2,3-dioxygenase (IDO). Interestingly, CD14 + HLA-DR^low^ monocytes in DLBCL patients also expressed higher levels of CD163, arginase and PD-L1 and inhibited CD8 + T cell proliferation [13]. Monocytic myeloid-derived suppressor cells (M-MDSCs) are early myeloid progenitors displaying strong immunosuppressive functions. Several studies demonstrated that a high number of M-MDSCs was associated with an unfavorable outcome in AML [14, 15], as well as in DLBCL [16]. In both Hodgkin and non-Hodgkin lymphoma patients, CD66b^+^CD33^dim^HLA-DR^−^ granulocytic MDSCs (PMN-MDSCs) are increased compared to healthy controls, display immunosuppressive functions and correlate with unfavorable outcomes [17]. Of note, in sepsis, granulocytic MDSCs (PMN-MDSCs) mostly, and to a lesser extent M-MDSCs, increase after the onset of infection [18], both contributing to T-cell dysfunction [19] although the detection of high levels of PMN-MDSCs months after sepsis suggested they may play a role in sepsis-induced immunosuppression and late nosocomial infections [18] (Fig. 1).

### Adaptive immune dysregulation

HMs can also participate in adaptive immune dysregulation, either by directly involving secondary lymphoid tissues (i.e., myeloma or chronic lymphocytic leukemia (CLL)) or the bone marrow niche (acute leukemias), or by altering adaptive immune cell functions through tumor-immune cell interactions or microenvironment signaling. It is known that hypogammaglobulinemia can affect patients with CLL and NHL, which increases the risk for severe bacterial infections. Hypogammaglobulinemia is commonly detected by serum protein electrophoresis. Soumerai et al. showed in a retrospective longitudinal study including 3.960 CLL patients and 13.232 NHL patients that increasing the number of IgG testing in this population improved hypogammaglobulinemia diagnosis and was associated with decreased risk of severe infection after adjustment in multivariable logistic regression [20]. Lymphoma can also affect adaptive immune cell phenotype. Hesterberg et al. showed in a recent study that B-cell lymphoma can induce aging-like phenotypes and age-related epigenetic reprogramming in young T cells, as well as accelerate aging in other tissues (aorta, kidney, liver…) [21]. It is well described in the literature that immune aging alters immune responses and increases the risk for acute infection [22]. In AML, Lasry et al. analyzed the remodeled bone marrow immune microenvironment in adult and pediatric patients and revealed that a subset of B cells, defined as atypical due to ITGAX, FCLR3 and FCLR5 expression although being both class-switched and non-class-switched B cells, was more often found in AML patients than in healthy controls, and was correlated with inflammation. Moreover, T cells were also affected by AML as CD8 + T cells were the most expanded clones and they displayed a continuum of activation, while T-cell clonal diversity was increased in AML patients with high inflammation [23]. In this study, the authors were also able to demonstrate that inflammation had a direct effect on patient outcomes, by defining an inflammation risk score which proved to be correlated with prognosis [23]. In B-cell ALL, it has been shown that the number of regulatory T cells (Tregs) was decreased compared to healthy controls, although their immunosuppressive function was increased and positively correlated to disease progression [24] (Fig. 1).

### The tumor-microbiome axis: chemotherapy- and malignancy-induced dysbiosis and impact on immune dysregulation

Since 2010, studies have consistently shown that chemotherapy profoundly alters the gut microbiota. In AML patients, multiple cohorts have reported a reduction in α-diversity, a loss of beneficial commensals, such as *Faecalibacterium*, and an overgrowth of potentially pathogenic bacteria-like *Enterococcus* [25–27]. These changes often persist long after treatment and are exacerbated during reinduction or salvage chemotherapy [28]. This dysbiosis is associated with chronic inflammation and may compromise intestinal barrier integrity. In a prospective cohort study of AML patients undergoing induction chemotherapy, Kapandji et al. [29] provide recent evidence linking severe neutropenic enterocolitis to intestinal barrier dysfunction and gut dysbiosis. The study identified marked shifts in microbiota composition, including reduced α-diversity and an overrepresentation of *Enterococcus faecalis*, alongside decreased butyrate levels. These microbial changes coincided with enterocyte injury, as indicated by lower citrulline levels, and the activation of inflammatory pathways, such as Janus kinase—Signal Transducer and Activator of Transcription (JAK-STAT) in colonic tissues. These findings highlight the critical role of microbiota integrity and mucosal immunity in the pathogenesis of neutropenic enterocolitis during chemotherapy.

Furthermore, emerging data suggest a mechanistic link between dysbiosis and leukemia progression. In a murine model of MLL-AF9 AML, Wang et al. demonstrated that reduced butyrate levels led to increased gut permeability, facilitating Lipopolysaccharide (LPS) translocation and promoting leukemic blast proliferation [30]. These findings emphasize the importance of the gut microbiota not only in chemotherapy toxicity but potentially in AML pathogenesis itself.

Immunotherapy may also considerably impact gut microbiota. In 48 patients with NHL or ALL treated with CAR-T cell infusion, Smith et al. demonstrated that high abundances of *Clostridia* in fecal microbiome at baseline before CAR-T cell infusion initiation was associated with day 100 complete response [31]. Finally, in allogenic stem cell transplant (HSCT), reduction in α-diversity is associated with mortality [32] or graft versus host disease (GVHD) [33]. Taken together, these data suggest that fecal microbiota transplant could be of interest in patients with HMs to improve immune anti-tumor response, decrease treatment toxicities, complications and mortality.

### Experimental models to study sepsis–HM interactions

Mouse models have provided critical insights into the complex interplay between cancer and sepsis, showing that the malignancy significantly alters the host’s response to severe infection. However, experimental studies specifically combining sepsis with leukemia or lymphoma are extremely limited. Indeed, most experimental studies studying the interaction between sepsis and malignancies have focused on solid tumors [34, 35]. Both the type of malignancy and the immune status of the host before sepsis induction (especially after chemotherapy and/or bone marrow suppression) may significantly affect the course of sepsis and cancer outcomes. Another important factor is the order in which cancer and sepsis occur. When tumor inoculation precedes sepsis in mice models, the septic process has been shown to slow or even inhibit tumor progression. In contrast, in models, where sepsis is performed before tumor inoculation, tumor progression is often accelerated, largely due to the accumulation of immunosuppressive cells (such as tumor-associated macrophages, MDSCs, and Tregs) in the tumor microenvironment [36, 37]. Another major consideration is the choice of the sepsis model itself, as different models reproduce distinct immune responses. For example, a model such as cecal ligation and puncture (CLP) is known to induce a state of immunosuppression that closely mimics the late phase of human sepsis, characterized by impaired lymphocyte function and expansion of immunosuppressive cell populations [38]. In contrast, single high-dose endotoxin injection induces acute hyperinflammation better reflecting the early phase of sepsis but not its immunosuppressive features [38]. Thus, translationally relevant insights into the interplay between sepsis and HMs depend on the choice of tumor model, the sequence of cancer and infection, and the type of sepsis model employed. Indeed, most mechanistic evidence derives from solid tumor models. The biology of HMs differs substantially, particularly with respect to the bone marrow microenvironment and, therefore, the relevance of these experimental findings to HMs. Nevertheless, it is important to keep in mind that mechanistic insights from these models should be considered hypothesis-generating rather than definitive evidence of causal effects in humans.

## Sepsis-induced modulation of haematological malignancies

### Effects of systemic inflammation on tumor progression

While no experimental study has directly investigated the potential contribution of sepsis to HMs’ development, clinical evidence suggests a possible link, keeping in mind that the current human evidence linking sepsis to HM development is largely observational, and therefore, causal inferences cannot be drawn. Moreover, registry-based studies reporting increased cancer incidence following sepsis may also be influenced by surveillance bias and survivor bias. In a large retrospective study of two million American patients, Liu et al. demonstrated that patients who have experienced sepsis have a higher risk of developing AML and MDS, although sepsis was inversely associated with diffuse large B-cell lymphoma or follicular lymphoma [39]. Chronic inflammation is already known to be independently associated with the development of AML [40, 41]. Moreover, chronic inflammatory or autoimmune diseases (i.e., systemic lupus erythematous, rheumatoid arthritis, Sjögren syndrome) are known risk factors for lymphoma development, aside from their immunosuppressive treatments [42, 43].

Epidemiological studies have also reported associations between clonal haematopoiesis of indeterminate potential (CHIP) or MDS and autoimmune manifestations, suggesting that acquired stem cell mutations may simultaneously impact HM risk and immune-mediated disease [44]. CHIP refers to the presence of somatic mutations in hematopoietic stem or progenitor cells in patients without a known hematologic malignancy. CHIP can lead to the expansion of clonal blood cells that may exhibit altered immune function. Given that these mutated cells can circulate and contribute to immune dysregulation, it is plausible that CHIP could influence susceptibility to severe infections, including sepsis. Therefore, CHIP represents a potential confounder in observational studies linking sepsis to subsequent HM, as pre-existing clonal expansions could both predispose to infection and increase future malignancy risk.

Although sepsis initially begins with an acute systemic inflammatory response, its long terms effects may mimic those of chronic inflammation, thereby supporting myeloid or lymphoblastic leukemic cell survival or the occurrence of point mutations which could participate in lymphoid malignancies development [45].

## Contribution of pro-inflammatory cytokines to haematological malignancies pathogenesis

**Fig. 2:**
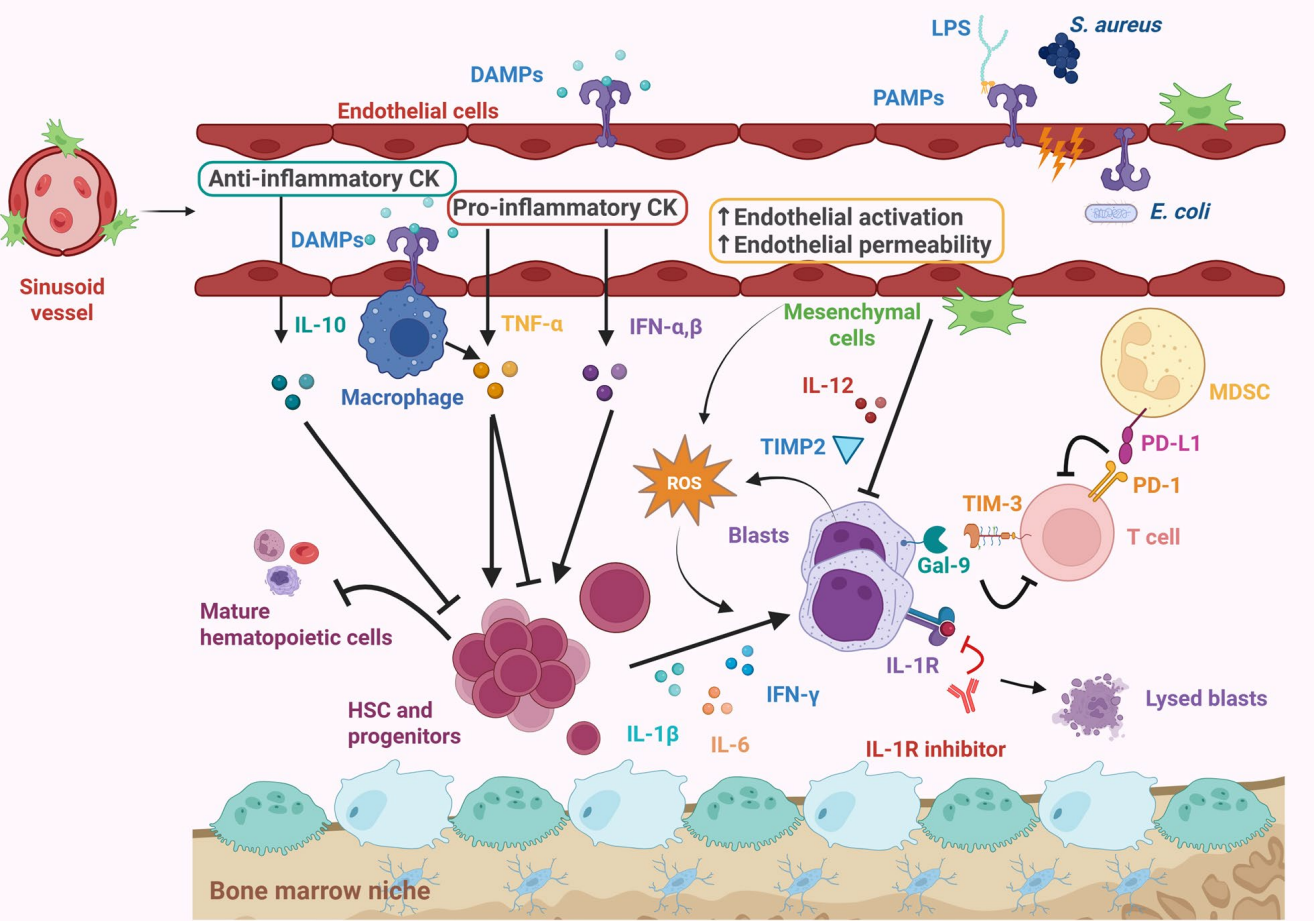
Impact of sepsis on bone marrow microenvironment: the example of acute myeloid leukemia. Through the recognition of DAMPs and PAMPs, sepsis induces increase of anti- and pro-inflammatory cytokines that may directly affect HSC expansion and differentiation. Endothelial cells during systemic inflammation and blasts increases the amount of reactive oxygen species (ROS), which cause DNA damages and genetic instability in HSCs. Sepsis may also suppress blast expansion through the secretions of tumor suppressive mediators (IL-12, TIMP2) by mesenchymal cells. IL-1β, IL-6 and IFN-γ contribute to AML blasts’ expansion at the expense of healthy progenitors. As an example, IL-1R has been suggested as a promising therapeutic target in AML. Sepsis-induced immunoparalysis may create a favouring microenvironment for tumor cells by increasing the amount of anti-inflammatory cytokines and immune checkpoint surface expression on immune cells. *DAMPs* Damage Associated Molecular Patterns, *PAMPs* Pathogen Associated Molecular Patterns, *LPS* Lipopolysaccharide, *CK* Cytokines, *IL* Interleukin, *IL-1R* Interleukin-1 Receptor *ROS* Reactive Oxygen Species, *IFN* Interferon, TNF Tumor Necrosis Factor, *TIMP2* Tissue inhibitor of metalloproteinases 2, *TIM-3* T-cell immunoglobulin and mucin-domain containing-3, *Gal-9* Galectin-9, *MDSC* myeloid-derived suppressor cell, *HSC* Hematopoietic stem cell, *PD-1* Program-death 1, *PD-L1* Program-death ligand 1. Created in BioRender. Pons, S. (2026) https://BioRender.com/es1e7hm

When sepsis occurs, large amounts of pro-inflammatory cytokines are released, such as Interleukin (IL)−1β, IL-6 and Tumor Necrosis Factor (TNF)-α, as well as anti-inflammatory cytokines like IL-10 (Fig. 2) [46]. B-cell NHL were at increased risk of occurrence in women with high levels of IL-10 and TNF [47]. Moreover, besides being a prognostic factor in Hodgkin lymphoma, it has been suggested that persons with genotypes associated with higher levels of IL-6 were more at risk of developing a Hodgkin lymphoma [48]. The IL-7/IL-7R axis may be involved in lymphoid leukemogenesis and treatment response [49]. Clinically, AML patients with elevated IL-10 and decreased IL-6 levels display improved survival [50]. Murine models have shown that prolonged exposure to pro-inflammatory cytokines can provide an oncogenic “second hit”, contributing to AML transformation [51]. For example, it has been demonstrated that IL-1β contributes to AML blasts’ expansion (around 70%) at the expense of healthy progenitors [52]. Similarly, IL-6 levels are elevated in the bone marrow during infection, increasing the pool of leukemic stem cells (LSC) [53], a process further amplified by the NF-kB activation downstream of TNF-α signaling [54]. These cytokines thus represent interesting therapeutic targets in AML. Indeed, blockade of IL-6 has been shown to improve survival by inhibiting the proliferation of MLL-rearranged Leukemic Stem Cells (LSCs) in MLL-AF9 murine models [55]. Similarly, the development of IL-1 inhibitors for aggressive MLL-rearranged leukemia represents an interesting approach to balance the amount of MLL oncogenic fusion proteins by modulating the Interleukin-1 Receptor Associated Kinase (IRAK)1/IRAK4 signaling pathways [56]. Ongoing clinical investigations are currently assessing the safety of Siltuximab (anti-IL-6) in AML patients (NCT05697510).

## Inflammation-induced modification of the bone marrow and lymphomagenesis

Inflammation exerts a dual effect on hematopoietic stem cells (HSC), which are highly sensitive to inflammatory signals, such as damage associated molecular patterns (DAMPs) or pathogen associated molecular patterns (PAMPs) [57]. Indeed, some studies have reported an increase of HSCs in the bone marrow following stimulation with LPS or *Pseudomonas aeruginosa*, at the cost of reduced functionality (reflected by impaired generation of granulocyte-monocyte progenitors, a diminished pool of innate immune-competent cells and a loss of transplant potential in murine models [58, 59]). In HSCs and progenitors, bis-(3’−5’)-cyclic dimeric guanosisne monophosphate (c-di-GMP), a known second messenger involved in numerous bacterial activities, has been identified as a signal-transducing PAMP influencing HSC and progenitor expansion and mobilization through extramedullary hematopoiesis and BM microenviroment modulation [60]. Conversely, systemic inflammation increases the amount of reactive oxygen species (ROS), which cause DNA damages and genetic instability in HSC, an important driver in AML’s leukemogenesis, especially in patients with predisposing mutations [61]. Furthermore, by modifying the components of the HSC niche (stromal cells, osteoblasts, endothelial cells), inflammation induces a loss of HSCs’ quiescence and contributes to their mobilization into the bloodstream [62].

Generalized inflammation induced by sepsis also affects the bone marrow microenvironment beyond HSCs and LSCs. Infections cause extensive remodelling of the bone marrow cellular populations, therefore, contributing to chemotherapy resistance [63]. For example, septic mouse models exhibit increased vascular permeability, endothelial proliferation and angiogenesis [64, 65] which could potentially contribute to myeloid cells egression into the bloodstream and especially blasts, worsening organs infiltration. Conversely, while mesenchymal stromal cells (MSCs) are generally known to support blast proliferation, Yu et al. has shown that that co-stimulation of MSCs and AML blasts with LPS could suppress blast expansion through the secretions of tumor suppressive mediators (IL-12, Fas-L, TIMP2) [66]. However, given the marked heterogeneity of MSC populations, these findings should be interpreted with caution, as dysregulation of MSC development has also been shown to facilitate AML and MDS progression [67].

It has also been shown that bacterial or viral infections could be responsible for lymphomagenesis. Indeed, *Helicobacter pylori* chronic infection is known to be associated with the development of gastric extranodal marginal zone-B lymphoma in mucosa-associated lymphoid tissue (MALT). Infiltration of the B-cell clone at the site of the gastritis may be present years before the onset of the MALT lymphoma [68]. Inflammatory environment can also drive cellular proliferation, favouring the acquisition of tumor-promoting mutations. In patients with Cutaneous T-cell lymphoma, Willerslev-Olsen et al. showed that Staphyloccocal enterotoxin A was able to activate STAT3, a well described oncogenic pathway when activated, and trigger IL-17 expression in primary malignant T cells, notably through IL-2 and IL-2 receptor [69]. B-cell receptor (BCR) signaling has been described as a mechanistic determinant of lymphoma development: BCR activation by different types of antigens (bacteria, virus, self-antigens) can, therefore, participate in tumorigenesis or lymphoma clinical progression [70].

### The duality of sepsis (Fig. 2)

One of the main challenges in assessing the impact of sepsis on HMs’ development is due to the concomitant occurrence of an initial excessive inflammation state and an immunosuppression state that can persist for months or years after the infection [71]. This dysregulated immune response induces a massive apoptosis of dendritic cells and the expansion of immature neutrophils with diminished recognition and phagocytic capabilities balanced by a persistent reduction in both the number and function of CD4 + and CD8 + lymphocytes and the expansion of immunosuppressive cells (Tregs and MDSCs) [72, 73]. As a potential result, the host could end up being more vulnerable to the recurrence of infections and/or the progression of underlying malignant diseases [36].

## Sepsis-induced upregulation of immune check-points and haematological malignancies’ progression

Immunosurveillance against tumor cells relies on a coordinated response between T CD8 +, Natural Killer (NK) cells and antigen-presenting (APC) cells. In contrast, sepsis-induced immunoparalysis may create a favouring microenvironment for tumor cells by increasing the amount of anti-inflammatory cytokines and immune checkpoint surface expression on immune cells, such as Program-death 1 (PD-1) and Program-death ligand 1 and 2 (PD-L1 and 2), Cytotoxic T-Lymphocyte-Associated protein-4 (CTLA-4), T-cell immunoglobulin and mucin-domain containing-3 (TIM-3) and Lymphocyte-activation gene 3 (LAG-3) [74]. For example, TIM-3 binds to galectin-9 expressed on AML blasts, inducing T-cell anergy through multiple downstream signaling pathways, such as MAPK/ERK, AKT and PI3K [75]. In diffuse large B-cell lymphoma, high levels of LAG-3 and PD-1 co-expression are associated with shorter survival and poorer prognosis, while inhibition of either or both of them improved CD8 + T-cell function and tumor cell apoptosis [76].

This contributes to immune tolerance and supresses adaptive immune responses, thereby facilitating for blasts’ or tumor cells’ proliferation [77].

## Immunosuppressive cell networks supporting tumor development

In mice, it has been demonstrated that the occurrence of systemic infection followed by tumor engraftment sometimes accelerates tumor growth [78]. This effect is largely mediated by the accumulation of PMN-MDSCs activated through the Toll-Like Receptor (TLR)−4/Myd88 signaling pathway [78], although the precise role of TLRs in tumorigenesis remains controversial [79]. MDSCs exert potent immunosuppressive effects by producing enzymes, such as arginase-1 and inducible nitric oxide synthase, which impair CD8⁺ T-cell proliferation and cytotoxicity [80]. They also inhibit CD4 + T cell and NK cell activation via PD-L1 expression, secrete regulatory cytokines (IL-10, TGF-β) [81] and promote Treg expansion cells through the secretion of IDO. Notably, an IDO1-related immune gene signature has recently been shown to predict survival in AML patients [82]. Finally, by secreting arginase-2, MDSCs facilitate the polarization of macrophages from M1 toward M2 phenotype, thereby reinforcing immunosuppression and promoting angiogenesis. In the context of AML, M2 macrophages promote immune evasion and provide a supportive niche for leukemic blasts, enhancing their survival and proliferation. They also contribute to angiogenesis and chemoresistance, thereby reinforcing disease progression [83].

The phenotype and function of NK cells, which trigger a non-specific innate immune response against cancer, may be also altered by sepsis [84]. In a recent paper, Rodriguez-Sevilla et al. demonstrated that MDS HSCs could escape immune surveillance due to dysfunctional and exhausted NK cells displaying reduced cytotoxic capacities against tumor cells [85], suggesting sepsis-induced NK cell alterations could participate in tumorigenesis in certain contexts.

## Conclusion

Both sepsis and HMs profoundly and durably impact innate and adaptive immune responses, which may directly influence their progression. Multiple cell types, molecular pathways, cytokines are involved and participate in the immune interplay between these two diseases. A better understanding of the multiple mechanisms involved in HMs that lead to increased risk of sepsis, as well as how can sepsis affect HM development and outcome is needed to improve HMs care and prognosis. Fundamental and translational studies are warranted as they may pave the way to the development of preventive or therapeutic strategies in HMs patients.

## Data Availability

All data discussed in this review are derived from publicly available sources cited in the reference list. No new datasets were generated or analyzed for this study.

## Declarations

### Artificial intelligence statement

The authors disclose the use of ChatGPT-4o to improve the style and readability of the English manuscript.

### Take-home message

Both sepsis and haematological malignancies profoundly and durably impact innate and adaptive immune responses, which may directly influence their progression. Multiple cell types, molecular pathways, cytokines are involved and participate in the immune interplay between these two diseases.

### A 140-character Tweet

Sepsis and haematological malignancies profoundly impact innate and adaptive immune responses, which may influence their progression.
